# Health in Conflict Zones: Analyzing Inequalities in Mental Health in Colombian Conflict-Affected Territories

**DOI:** 10.3389/ijph.2021.595311

**Published:** 2021-05-17

**Authors:** Sebastián León-Giraldo, Germán Casas, Juan Sebastian Cuervo-Sanchez, Catalina González-Uribe, Oscar Bernal, Rodrigo Moreno-Serra, Marc Suhrcke

**Affiliations:** ^1^ Interdisciplinary Centre of Development Studies, Universidad de Los Andes, Bogotá, Colombia; ^2^ Alberto Lleras Camargo School of Government, Universidad de Los Andes, Bogotá, Colombia; ^3^ School of Medicine, Universidad de Los Andes, Bogotá, Colombia; ^4^ Fundación Santa Fe de Bogotá University Hospital, Bogotá, Colombia; ^5^ Centre for Health Economics, University of York, York, United Kingdom; ^6^ Luxembourg, Institute of Socio‐Economic Research (LISER), Luxembourg

**Keywords:** Colombia, mental health, socio-economic inequalities, decomposition analysis, conflict

## Abstract

**Objectives:** Colombia’s civil conflict and persistent socio-economic disparities have contributed to mental health inequalities in conflict-affected territories. We explore the magnitude of mental health inequalities, contributing socio-economic factors, and sociodemographic characteristics that explain these differences.

**Methods:** The study draws on data collected in 2018, using the household survey *Conflicto, Paz y Salud* (CONPAS) applied to 1,309 households in Meta, Colombia. Logistic regression and decomposition analysis were used to analyze the risk of mental health disorders, measured with the Self-Reporting Questionnaire -20 (SRQ-20).

**Results:** Individuals with lower socio-economic status are at a higher risk for mental health disorders. Forced displacement accounts for 31% of the measured mental health inequalities. Disparities in employment, education level, disability and conflict incidence between municipalities are other contributing factors. Women and people with disabilities are respectively 2.3 and 1.2 times more prone to present a mental health disorder.

**Conclusion:** It is necessary to tackle the identified risk factors and sociodemographic circumstances that contribute to mental health inequalities in conflict-affected territories, as these hinder adequate/equitable access to mental health services.

## Introduction

Mental health is a global development issue in conflict-affected territories [1]. Conflict may increase the chances of experiencing mental disorders, such as depression, anxiety, and psychosomatic disorders [2]. Reports around the world show a high prevalence of negative mental health outcomes as a consequence of war [3]. A study on the Rwandan genocide reports a 24.8% prevalence of post-traumatic stress disorder (PTSD) symptoms in 2,091 subjects [4]. Similarly, a study on the 20th-century Guatemalan civil war reports anxiety symptoms in 54.4%, depression symptoms in 38.8%, and PTSD in 11.8% of refugees, 20 years after the civil conflict [5]. The violence experienced during civil armed conflicts tends to persist over time, an issue which produces sustained mental health problems among conflict-affected populations for several years. This leads not only to difficulties in long-term recovery but also to the “cultural normalizationˮ of this phenomenon [6].

Colombia’s armed conflict is one of the longest-lasting civil armed conflicts in the world. It has lasted for more than 50 years and has caused approximately 220,000 deaths (1958–2012), 1,982 massacres, 27,023 kidnappings (1970–2010), 25,007 forced disappearances, and more than 4,744,046 internally displaced people [7]. The Colombian conflict has led to a high prevalence of mental disorders in the affected territories. A small-scale study (N = 109) in the country found a high prevalence of mental disorders, specifically PTSD symptoms (88%), anxiety (44%), and depression (41%), in people who have experienced internal displacement in conflict-affected territories [8].

The armed conflict in Colombia has simultaneously perpetuated direct conflict-related violence, associated with higher anxiety-related psychopathologies, as well as non-conflict violence, such as a higher prevalence of substance abuse and domestic violence [9]. Additionally, conflict-related violence has affected access to healthcare for the most vulnerable populations who have experienced the hardships of war [10]. Therefore, it is necessary to identify contributing factors that sustain negative mental health outcomes in long-lasting civil conflicts [11].

Mental health outcomes may be characterized by the influence of socio-economic circumstances on an individual’s health. The most influential conceptual model, the *Social Determinants of Health* (SDH), defines these determinants as “the circumstances in which people are born, grow up, live, work, and age and the systems put in place to deal with illness” [12]. These socio-economic factors establish the everyday risks or protective factors to which people are exposed and which, altogether, determine health. Consequently, it is important to identify those circumstances that most influence health in conflict-affected territories.

Poverty is one of the most studied social determinants of mental health. High levels of poverty in low-to middle-income countries tend to be associated with an increased incidence of mental disorders due to financial and economic stress [13]. At the same time, mental health issues affect social engagement, labor, and work productivity [14]. A lower income also results in exclusion from adequate health services [15], leading to broader negative mental health consequences. In the long run, these circumstances increase disparities among families and territories.

Poor quality and access to educational services, as well as occupational social class differences, tend to increase disparities among populations [16]. Individuals with low socio-economic status (SES) tend to be at a higher risk of developing mental health disorders [17]. In regions such as Latin America which, historically, have been characterized by high levels of socio-economic inequality, this leads to reduced access to mental health services and inequalities in mental health [18].

Tackling inequalities in mental health can guarantee a better quality of life for the population [19]. The prevalence of mental health disorders may produce further inequalities in other areas of the individual’s life or increase health-risk behaviors [20]. Furthermore, these disparities tend to become more persistent over time, due to inequalities in healthcare provision and greater barriers to accessing mental healthcare services [21]. The prevalence of these disorders in people at lower income levels increases the social gap which, in the long run, may contribute to poverty persistence [22]. Establishing the relevant barriers, differences, or sociodemographic characteristics that contribute to these inequalities in mental health can improve access to adequate health services [23].

The present study determines which sociodemographic factors account for the inequalities in mental health in a territory that was highly affected by the Colombian conflict in 2014 by analyzing the social determinants of health. The study uses information provided by the CONPAS survey conducted in Meta, Colombia in 2018, but which includes retrospective information about mental health outcomes for 2014.

The following section presents our methodological approach, followed by our main results. We conclude with a discussion of the main implications and the conclusions that can be drawn from our results.

## Methods

The CONPAS survey is representative at the level of conflict incidence of the municipality, measured using the scale developed by the Conflict Analysis Resource Center (CERAC). The population under analysis were households or habitual residents living in Meta, Colombia. The survey sample was selected through a probabilistic multistage sampling. First, random square blocks were selected in each municipality, then specific residences were selected and, finally, for each residence, one single household. The head of the household or a key informant was identified to conduct the survey.

First, we performed a descriptive analysis to conduct appropriate data cleaning procedures. Some categorical variables were regrouped to have a minimum number of observations per category for statistical analysis. We excluded people in our sample who, in 2014, were minors or not living in Meta, to focus our analysis in this specific conflict-affected region. This process left us with a sample size of 1,089 individuals.

We conducted a logistic regression to measure the importance of several sociodemographic variables on mental health outcomes. Then, we performed a decomposition analysis [24] to disaggregate mental health inequities as a function of inequities of socioeconomic determinants. Households were classified by socioeconomic levels using the Household Wealth Index (HWI) [25]. The HWI was used to measure the inequality in the distribution of mental health disorders in our sample. The wealth index for a specific household is defined as

$${\text{HWI}}_{i}=\alpha_{1}\left(\frac{x_{1}-{\bar{x}}_{1}}{s_{1}}\right)+\alpha_{2}\left(\frac{x_{2}-\bar{x_{2}}}{s_{2}}\right)+\ldots \alpha_{k}\left(\frac{x_{k}-\bar{x_{k}}}{s_{k}}\right)\;,$$ (1)

where *x* are the independent binary variables measuring the ownership of a specific asset related to wealth, xbar is the mean of this variable, *s* is its standard deviation, and alpha are the relative weights of each variable. For the x variables, we used specific questions that measured access to different household assets. The weight of each variable was obtained using Principal Component Analysis. Finally, using the HWI, we divided our sample by poverty quintiles.

We used the Self Report Questionnaire-20 (SRQ-20), an instrument developed by the World Health Organization, to measure mental health outcomes [26]. If a person answers “yesˮ to eight or more questions on the SRQ-20, he/she presents a positive tendency (SRQ+) of presenting a mental health disorder.

We then constructed the Health Concentration Index (HCI), using the HWI and our mental health indicator (SRQ+) [24]. The HCI measures the inequality in the distribution of mental health disorders in a population and ranges between −1 and 1, where a negative value represents an unequal distribution against poorer individuals, 0 represents a perfect distribution, and a positive value represents inequality against richer individuals. The HCI is defined as the ratio of the area between the concentration curve and the line of equality against the total area under the line of equality:

$$\text{HCI}=\;\frac{2\mathrm{cov}\left(Y,R\right)}{\mu_{y}},$$ (2)

where *Y* is the health variable (SRQ+), *µ*_*y*_ is its mean, and *R* is the person’s rank (or position) in the income distribution. The ranking variable, which ranges from 0 to 1, classifies individuals according to socioeconomic status, based on the HWI index.

However, since our explanatory variable is dichotomous (0, 1), an appropriate correction must be applied to scale the range of the index to its possible values. Wagstaff [27] proposed a variation of Eq. 2 for binary outcome variables as follows:

$${\text{HCI}}_{N}=\frac{\text{HCI}}{1-\mu_{y}\;},$$ (3)

with this transformation, the range of our HCI moves from $\mu_{y}-1$ to $1-\;\mu_{y}$ , ensuring that the HCI can be interpreted between the values of a standard concentration index.

We can calculate the individual contribution of socioeconomic determinants in explaining mental health inequalities through decomposition analysis. This method measures the extent to which inequalities in mental health, measured by the HCI index, can be attributed to disparities among specific independent variables. For our analysis, explanatory variables included several sociodemographic variables that have been associated with differences in mental health outcomes in the academic literature.

To determine the relative importance of our independent variables in explaining the inequalities in mental health, we applied a probit model. Independent concentration indices ( $CI_{X}$ ) were calculated for our explanatory variables using Eq. 4:

$$CI_{X}=\;\frac{2\mathrm{cov}\left(X,R\right)}{\mu_{x}},$$ (4)

where X is the independent variable, $\mu_{x}$ is its mean, and R is the ranking variable of individuals categorized by income levels. These concentration indices measure the existing inequalities in the distribution of the independent variable among different socioeconomic levels.

Using the marginal effects of the probit model, we then estimated the weight or relative importance of each independent variable for describing our explanatory variable, namely, the SRQ indicator.

$${\text{Weight}}_{x}=\beta_{X}\frac{\mu_{x}}{\mu_{y}\;},$$ (5)

where $\beta_{X}$ is the marginal effect of our probit estimation and $\mu_{x}$ and $\mu_{y}$ are the means of our independent and dependent variables. The weight measures the relative importance of this independent variable in explaining the overall HCI of mental health. The contribution of an independent variable to the HCI is estimated as:

$$\text{Contribution to }C_{x}={\text{Weight}}_{x}*CI_{X}.$$ (6)

Finally, we can estimate the percentage or ratio by which inequalities in mental health disorders, the HCI value, would be reduced if inequalities in a specific independent variable are reduced to zero by:

$$\text{Percentage reduction of HCI }{\text{by}}_{x}=\frac{\text{Contribution to }CI_{x}}{\text{HCI}}.$$ (7)

## Results

Table 1 presents the summary statistics of the sample (N = 1089). As shown, 53% of the sample is composed of women. The age range most represented is 18–44 years, accounting for 51.8% of the total population. Up to 39.3% of the sample lives in rural areas, whereas 13.1 and 44.2% live in municipalities with high and low armed conflict impacts, respectively. In 2018, the year when the CONPAS survey was applied, 43% of the sample reported themselves as people currently experiencing internal displacement. Finally, 15.5% of the population has a positive tendency to present mental health disorders, as measured by the SRQ-20 in 2014.

**TABLE 1:** Summary statistics–sample of 2014 for survey *Conflicto Paz y Salud*.

Sample: CONPAS, 2014 (N = 1089)	N	%
Gender		
Men	508	47.0
Women	581	53.0
Age group		
18–44	564	51.8
45–64	412	37.8
65+	113	10.4
Household location		
Urban area	661	60.7
Rural areas	428	39.3
CERAC conflict classification		
Capital city	282	25.9
Highly affected municipality	143	13.1
Not affected municipality	183	16.8
Lowly affected municipality	481	44.2
Number of internally displaced people (IDP)–reported in 2018	468	43.0
SRQ+ mental health indicator	169	15.5

Source: Prepared by authors based on CONPAS 2014. CERAC conflict classification: Classification used by the Conflict Analysis Resource Center (CERAC) to measure conflict incidence in a municipality. SRQ+: Possible mental health disorder measured by the Self Report Questionnaire.

### Factors Explaining Tendencies to Experience Mental Health Disorders

We conducted a logistic regression to identify which sociodemographic variables are most associated with a positive tendency to present mental illness (SRQ+). Table 2 presents the differences between people who have SRQ+ and those who do not.

**TABLE 2:** Logistic regression–Self-Report Questionnaire + and sociodemographic variables.

Variable	SRQ−		SRQ+		Or (95%)
	N	% sample	N	% sample	
Total					
Internally displaced (*p*-value: 0.006)					
1. No	554	60.2	67	39.6	1
2. Yes	366	39.8	102	60.4	1.90**
Age group (*p*-value: 0.097)					
1.18–44 years	485	52.7	79	46.75	1
2.45–64 years	343	37.2	69	40.83	0.84
3.65 or more	92	10.0	21	12.43	0.61
Sex (*p*-value: <0.001)					
1. Men	456	49.6	52	30.8	1
2. Women	464	50.4	117	69.2	2.30**
Household location (*p*-value: 0.316)					
1. Urban area	559	60.8	102	60.3	1
2. Rural area	361	39.2	67	39.7	0.88
Job type (*p*-value: 0.217)					
1. Working (formal)	211	22.9	30	17.7	1
2. Working (informal)	646	70.2	129	76.3	1.17
3. Out of the labor force	63	6.9	10	5.9	0.79
CERAC conflict classification (*p*-value: 0.009)					
1. Highly affected municipality	123	13.4	20	11.8	0.89
2. Lowly affected municipality	386	42.0	95	56.2	1.34
3. Not affected municipality	157	17.1	26	15.38	1.46
4. Capital city	254	27.6	28	16.6	1
Ethnicity (*p*-value: 0.330)					
1. Non-minority	727	79.0	124	73.4	1
2. Minority	193	21.0	45	26.6	0.99
Education (*p*-value: <0.001)					
1. None	48	5.2	17	10.1	2.06
2. Primary school	401	43.6	92	54.4	1.32
3. Secondary school	312	33.9	38	22.5	0.83
4. Higher education	159	17.3	22	13.0	
WHODAS score (*p*-value: <0.001)	—	—	—	—	1.19**

Source: Prepared by the authors based on CONPAS 2014 (Note: *p < 0.05, **p < 0.01 –SMMLV: Colombian Minimum Wage–[616.000 COP (257 USD) in 2014] (N: 1089) CERAC: conflict analysis resource center WHODAS: world health organization disability assessment schedule.

Experiencing internal displacement increases the odds of experiencing a mental health disorder by 1.9 times in comparison to those who are not displaced. Moreover, women are 2.3 times more likely to experience a mental health disorder compared to men who have similar characteristics. Finally, people who have any sort of disability, measured by the World Health Organization Disability Assessment Schedule (WHODAS), are 1.2 times more prone to present a mental health disorder.

Although a logistic regression quantifies the change in the *odds* of presenting tendencies of mental health disorders when an individual presents a certain sociodemographic characteristic, this analysis does not allow to measure inequalities in the distribution of mental health outcomes within a population group. Moreover, it prevents us from estimating the extent to which inequalities in mental health outcomes can be explained by inequalities in other determinants. Because of this, we performed a decomposition analysis of mental health inequalities among the CONPAS population.

### Inequality in the Tendency of Presenting Mental Health Disorders

**FIGURE 1:**
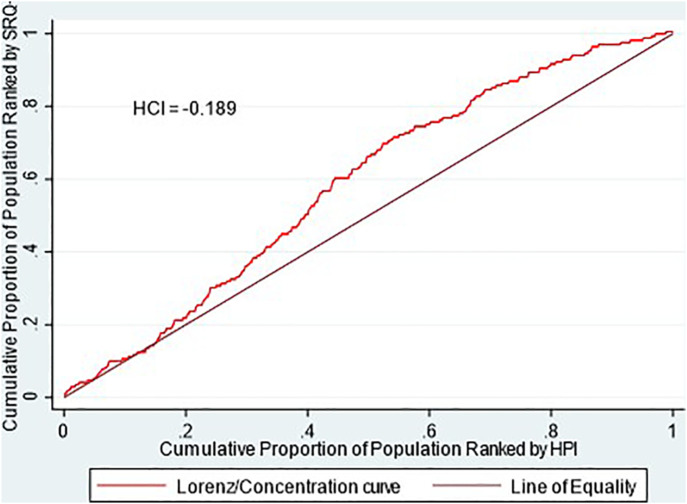
Concentration curve 2014–SRQ+. Source: prepared by the authors based on CONPAS 2014. SRQ+: possible mental health disorder measured with Self Report Questionnaire. HPI: household poverty index. HCI: health concentration index.

Using the HWI index and the distribution of SRQ+ cases, we constructed a concentration curve for this mental health indicator in 2014. The results are shown in Figure 1.

A concentration curve above the line of perfect equality is an indicator that SRQ+ is unevenly distributed among the population at different levels of income. The curve for 2014 and the negative HCI of −0.189 (rounded to −0.19) indicates that mental health disorders are disproportionally concentrated among the CONPAS survey population in 2014. A negative HCI indicates that, on average, people at lower socioeconomic levels have higher tendencies of presenting mental health disorders than people at higher income levels. The bulk of the inequality, in this case, is concentrated in people who have medium income levels. However, the poorest members of CONPAS 2014 show a fair distribution of the number of cases of mental health disorders.

### Decomposition Analysis—CONPAS 2014

Using the variables included above in the logistic regression as independent factors, we performed a decomposition analysis of the health concentration index for mental health (Figure 1).

A social determinant can contribute to mental health inequality in one of two ways [28]: first, if the determinant has a high prevalence among people with low income (CI is negative) and is associated with higher tendencies to present mental health disorders (marginal effect Beta is positive). These represent socio-economic risk factors mostly found in people at lower income levels. Second, if the determinant has a high prevalence among people with high-income levels (CI is positive) and is associated with lower chances of presenting mental health disorders (Beta is negative). These are protective factors found mostly in people with higher socio-economic status. The percentage of contribution shown on the last column of Table 3 indicates the magnitude by which inequalities in mental health outcomes could be reduced if the inequalities in that specific determinant were reduced to zero, if the effect of these determinants on mental health outcome is causal.

**TABLE 3:** Inequalities in mental health—decomposition analysis–*Conflicto Paz y Salud* 2014.

	Coefficient	Mean	Concentration index (C)	Elasticity	Cont. C	% of cont
Health concentration index (HCI)	NA		−0.19	NA		
Internally displaced						
1. No	Baseline					
2. Yes	0.07	0.43	−0.32	0.19		31.18%
Age group						
1.18–44 years	Baseline					
2.45–64 years	−0.02	0.38	−0.05	−0.05		−3.87%
3.65 or more	−0.05	0.10	−0.03	−0.03		−1.83%
Sex						
1. Men	Baseline					
2. Women	0.08	0.53	0.27	0.27		−21.20%
Household location						
1. Urban	Baseline					
2. Rural	−0.14	0.39	−0.67	−0.03		−12.20%
Job type						
1. Working (formal)	Baseline					
2. Working (informal)	0.01	0.71	−0.43	0.07	−0.03	14.80%
3. Out of the labor force	−0.02	0.07	0.34	−0.01	−0.00	1.50%
CERAC conflict classification						
1. Capital city	Baseline					
2. Highly affected municipality	−0.01	0.13	−0.45	−0.01	0.00	−1.35%
3. Not affected	0.04	0.17	0.21	0.04	0.01	−4.98%
4. Lowly affected municipality	0.03	0.44	−0.27	0.10	−0.03	13.72%
Ethnicity						
1. Non-minority	Baseline					
2. Minority	−0.00	−0.22	−0.23	−0.00	0.00	−0.4%
Education level						
1. None	0.07	0.06	−0.39	0.03	−0.01	5.48%
2. Primary school	0.03	0.45	−0.41	0.09	−0.04	18.97%
3. Secondary school	−0.01	−0.32	0.24	−0.03	−0.01	3.67%
4. Higher education	Baseline					
WHODAS	0.02	3.16	−0.15	0.36	−0.05	27.50%

Source: Prepared by the authors based on CONPAS 2014 CERAC: conflict analysis resource center WHODAS: world health organization disability assessment schedule.

Results show that experiencing internal displacement significantly contributes to mental health inequalities (31%). Displacement is highly concentrated in people with lower socio-economic status (−0.32) and people experiencing internal displacement are 0.07 times more prone to experience a mental health disorder in comparison to those who are not displaced. Ethnicity does not contribute to mental health inequalities as differences in the chances of experiencing mental health disorders between majority and minority ethnic groups are almost the same (−0.00). Even though women are much more prone to experience mental health disorders in comparison to men, gender does not contribute to mental health inequalities along different socio-economic groups since gender distribution is similar along socio-economic levels. Living in rural vs. urban areas does not contribute to mental health inequalities. Even though people in urban areas are more prone to experience a mental health disorder in comparison to rural areas (−0.14), the population in urban areas is constituted mostly by people of higher income levels. Living in rural areas, for lower-income populations, is acting, in this case, as a protective factor. Finally, experiencing any type of disability contributes to mental health inequalities (27%). Higher disability levels are found in people at lower-income groups (−0.15) and suffering any degree of disability increases the chances of experiencing a mental health disorder by a factor of 0.02.

For categorical explanatory variables, it is better to analyze marginal effects and concentration indexes individually, since the sign of the percentage of contribution in column 4 of Table 3 is sensitive to the base category we use. Age contributes to mental health inequalities since middle-age population groups have higher chances of experiencing mental health disorders, in comparison to older population groups, and this group is mostly found in lower socio-economic levels (CI: −0.05). Education highly contributes to mental health inequalities. People with no education and those with only a primary level have higher chances of experiencing mental health disorders in comparison to those with higher education levels (0.043 and 0.082 respectively). Having no education almost doubles the chances of experiencing a mental health disorder in comparison to people with primary education. These people are also highly concentrated in the lowest socio-economic groups, with a CI of −0.39 for no education and a CI of −0.41 for primary level education. People working in informal jobs are mostly found in the lower socio-economic groups (CI: −0.43) and have higher chances of experiencing mental health disorders in comparison to people with formal jobs.

People living in municipalities with no conflict, or municipalities that are low affected have higher tendencies to present mental health disorders in comparison to people living in the capital city or in highly affected territories. Living in the capital city increases mental health inequalities. This determinant acts as a protective factor in favor of higher-income groups, who are more concentrated in the capital city (CI: 0.456) and have lower chances of experiencing mental health disorders in comparison to those living in other Meta municipalities (0.03). Finally, living in a municipality with low conflict incidence increases mental health inequalities. People living in these territories, mostly of lower socio-economic levels (CI: −0.27), have an increased chance of having a mental disorder by nearly 0.04.

## Discussion

### Main Conclusions

The results of the analysis of the CONPAS 2014 survey data show that the tendencies to present mental health disorders are unevenly distributed among the population of Meta. Specifically, disparities tend to be larger for individuals at low- and middle-income levels. The overall inequity measured by the HCI is equal to −0.19, indicating that inequalities are more pronounced at low-income levels.

Decomposition analysis shows several structural and intermediate determinants important for explaining mental health inequalities. Internal displacement is particularly important as it tends to be more common in people already living under socio-economic difficulties. Displacement obligates individuals to leave their households, their support networks, and assets. Displaced individuals generally arrive to places where health circumstances are inadequate, such as poor neighbourhoods with high levels of crime, drug trafficking and other social issues, which negatively influence mental health outcomes.

Results show that a municipality’s conflict incidence level partially contributes to mental health inequalities. Multiple mechanisms could explain these results, most of them related to structural inequalities between territories. People in conflict-related environments are not only more exposed to violence, which influences mental health, but also may have limited access to mental health services. Capital cities may be in an adequate position to provide more stable health services and to have access to more skilled health practitioners. These inequalities are structural and context-related, and usually out of an individual’s control, making them especially difficult to tackle. However, mental health inequalities in highly affected territories may not be discernible due to general negative health outcomes in all population groups or generally positive mental health outcomes in no conflict territories.

Urban zones may be exposed to additional stressors like gang violence, sound contamination and other circumstances that can influence health outcomes negatively, in comparison to rural areas. Even though rural areas tend to concentrate people of lower socio-economic groups, mental health outcomes are not systematically worse. This may indicate several implicit benefits of living in these territories, such as lifestyle-related factors or stronger relationships among peers.

Results show that social and economic circumstances present the greatest contribution to mental health inequalities. Our data shows that people working in formal jobs and with higher education levels are not only of higher socio-economic groups but also have lower chances of experiencing a mental health disorder. Mechanisms explaining the importance of work status and education determinants may be explained primarily through two mechanisms: their influence on income and on opportunities. Having a formal job may lead to greater financial stability and reduce stress from possible financial difficulties. Education usually contributes to better income streams but, most importantly, generates more opportunities in life and work, which may improve living standards, and simultaneously, reduce health risk behaviors and negative mental health outcomes.

Individual determinants, those inherent to a person, such as age and ethnicity, are also important, but our analysis showed that they tend to be less determinant for explaining mental health inequalities. Younger population groups have higher chances of experiencing mental health disorders and are especially concentrated in lower socio-economic groups. Our results do not show significant differences between ethnic groups which may indicate that violence or social difficulties are not affecting a specific ethnic group or community systematically.

Our results show that women have a higher probability of experiencing a mental health disorder in comparison to men, a conclusion that is consistent with the international literature [23, 29]. However, as gender distribution is relatively similar between socioeconomic groups, this variable does not explain overall mental health inequalities between income groups in these territories. In Colombia, and specifically in conflict-affected regions, negative health outcomes among women have been associated mostly to gender-based violence originating from conflict [34]. Examples include violence against a spouse or partner (sometimes leading to death) by armed groups, rape, or forced abortion, and forced recruitment of children at very young ages [34]. However, since gender distribution is relatively similar between socioeconomic groups in our sample, this variable does not explain the overall mental health inequalities between income groups found in our study. However, because as the conflict may exacerbate pre-existing vulnerabilities among women, such as intimate partner violence, specific programs targeted to promote women’s mental health and recovery are important to minimize the long-run mental health consequences of the conflict for this group, especially in highly affected territories.

### Comparison With Previous International and National Studies

The results show similarities to national and international studies evaluating mental health inequalities. Cuartas et al. [23] performed a similar decomposition analysis with the National Mental Health Survey of Colombia in 2015, finding an HCI of −0.12 with a nationally representative population. Our results show higher inequality levels (HCI: −0.19), a difference that may be ascribed to our study concentrating on a territory that has been historically more affected by the armed conflict.

Sociodemographic differences that explain mental health inequalities are consistent with previous national and international studies. Morasae et al. [29] evaluated the mental health disparities in Iran’s capital Tehran, where the contribution of education status represented 13.4% of the total mental health inequalities, which is somewhat lower compared to our study (approximately 18.9%). Although education and employment status contributed to our general overall inequality levels, the contribution of internal displacement (31%) demonstrates that conflict may further increment inequalities in mental health.

Displacement and living in a municipality affected by armed conflict contribute in a certain degree to mental health inequalities. Displaced populations in Colombia experience difficulties in generating sustainable income, face greater social risk, and are forced to come up with diverse coping strategies to solve everyday problems [30]. Territories exposed to armed conflict in Colombia experience difficulties in medical health provision, frequent attacks against medical mission personnel, and limitations in the implementation of sustainable medical healthcare systems [10]. Nevertheless, in territories with high exposure to conflict, mental health outcomes may be negative in all population groups, which might explain way inequalities in health outcomes could not be discernible between socio-economic levels.

### Strengths and Weaknesses

Our study contributes to further the discussion on the impact and consequences of armed conflict in mental health outcomes. Additionally, it exhibits risk factors and sociodemographic circumstances that may increase mental health disparities. Concentrating on a territory highly affected by the armed conflict enabled us to establish the differences and determinants of mental health disorders and disparities that may not be easily discernible, considering only the national mental health average outcomes. This highlights the importance of analyzing mental health in territories at different conflict intensity levels.

A key contribution of our study is our analysis of the influence of people’s home territories on mental health outcomes, commonly known as neighborhood effects [31]. International research on conflict and mental health has focused mostly on the consequences of direct conflict violence, an approach that tends to overemphasize the impact of direct war exposure in mental health [32]. Our study offers news research perspectives on the impact of conflict intensity in mental health, not only in specific population groups (victims, soldiers etc.) or using case studies, an approach common in recent literature, but through a large descriptive survey study of a population.

Our analysis was conducted at the household level in Meta. Therefore, it may not reflect the circumstances of those who live in the most stressful and difficult economic situations, particularly those who do not own a house or have a permanent residence. This limitation may be concealing more profound mental health disparities in the poorest population.

### Final Remarks

Mental health is a global development issue and is one of the invisible problems in international development [1]. Social inequities also comprise one of the most prevalent problems of Latin American countries. These problems, combined with the presence of armed conflict in especially vulnerable communities, may increase the prevalence of mental health disorders and simultaneously lead to difficulties in recovery, treatment, and long-term quality of life. Political importance, public policy interventions, and prioritization must be conducted to guarantee that these populations find adequate mental health services and have psychosocial accompaniment to improve their general wellbeing. Closing this gap adequately and incorporating social circumstances into mental health programs is an important way, not only to improve general wellbeing but to simultaneously consider that the statement “no health without mental health” [33] is even more relevant in conflict-affected territories.

## Data Availability

The datasets presented in this study can be found in online repositories. The names of the repository/repositories and accession number(s) can be found below: Universidad de Los Andes http://hdl.handle.net/1992/45861.
